# Interactive mHealth Applications for Caregiver Training in Urinary Catheterization: A Scoping Review

**DOI:** 10.3390/nursrep16060194

**Published:** 2026-06-05

**Authors:** Hortência Fernandes, Layze Braz de Oliveira, Marília Duarte Valim, Herica Emilia Félix de Carvalho, Daniela Reis Joaquim de Freitas, André Luiz Silva Alvim, Daniel de Macedo Rocha, Aires Garcia dos Santos Júnior, Beatriz Maria Jorge, Inês Fronteira, Álvaro Francisco Lopes de Sousa

**Affiliations:** 1 Campus Três Lagoas, Federal University of Mato Grosso do Sul (UFMS), Três Lagoas 79070-900, MS, Brazil; h.fernandes@ufms.br (H.F.); daniel.macedo@ufms.br (D.d.M.R.); aires.junior@ufms.br (A.G.d.S.J.); beatriz_jorge@ufms.br (B.M.J.); 2 Department of Nursing, Federal University of Maranhão (UFMA), Pinheiro 65200-000, MA, Brazil; layze.braz@ufma.br; 3 Department of Nursing, Federal University of São Carlos (UFSCar), São Carlos 13565-905, SP, Brazil; marilia.duarte.valim@gmail.com; 4 Department of Nursing, State University of Maranhão (UEMA), Coroatá 65415-000, MA, Brazil; hericacarvalho@professor.uema.br; 5 Department of Nursing, Federal University of Piauí (UFPI), Teresina 64049-550, PI, Brazil; danielarjfreitas@ufpi.edu.br; 6 Department of Nursing, Federal University of Juiz de Fora (UFJF), Juiz de Fora 36036-900, MG, Brazil; 7 Public Health Research Centre, Comprehensive Health Research Center (CHRC), REAL, NOVA University Lisbon, 1050-091 Lisbon, Portugal; inesfronteira@ensp.unl.pt

**Keywords:** mobile applications, caregivers, urinary catheterization, health education, digital health, scoping review, nursing care, nursing

## Abstract

**Background/Objectives:** Urinary catheterization is common across care settings, but safe management at home and during care transitions often depends on caregivers who receive limited and inconsistent training. Mobile health (mHealth) applications may support caregiver education and decision-making. This review mapped and synthesized evidence on interactive mobile applications for caregiver training in urinary catheterization and developed a conceptual framework to inform nursing practice. **Methods:** A scoping review was conducted according to Joanna Briggs Institute guidance and reported following PRISMA-ScR. Searches were performed in PubMed/MEDLINE, Scopus, Web of Science, and LILACS, with complementary grey literature searches. Studies evaluating interactive mobile applications for caregiver training in urinary catheterization were included. Data were extracted and synthesized descriptively and narratively. **Results:** Five studies published between 2020 and 2025 were included. Most were early-stage studies with small samples and heterogeneous designs. Interventions generally combined educational content with interactive features, such as decision-support tools, and less often behavioral strategies, including reminders and feedback. Outcomes mainly addressed knowledge, skills, and self-efficacy, while clinical outcomes, such as infection reduction, were rarely assessed. A conceptual framework was developed showing how intervention components may influence caregiver competence and care outcomes, moderated by contextual factors such as health literacy and digital access. **Conclusions:** Interactive mobile applications may represent a promising approach to support caregiver training and improve the safety of urinary catheter management. However, current evidence remains preliminary and limited.

## 1. Introduction

Urinary catheterization is a widely used clinical procedure across hospital, community, and long-term care settings, playing a central role in the management of urinary retention, neurogenic bladder dysfunction, perioperative care, and accurate monitoring of urinary output. Despite its clinical utility, inappropriate catheter management remains a major contributor to adverse events, including urethral trauma and catheter-associated urinary tract infections (CAUTIs), which are among the most prevalent healthcare-associated infections worldwide and impose substantial clinical and economic burdens on health systems, patients, and families [1,2]. In this context, the competence of those responsible for catheter insertion, maintenance, and monitoring, whether healthcare professionals or informal caregivers, emerges as a critical determinant of patient safety and quality of care.

Two primary modalities of urinary catheterization are commonly employed in both clinical and home-based settings: indwelling catheterization and intermittent catheterization. The former involves continuous bladder drainage through a catheter maintained in situ by a retention balloon, while the latter consists of periodic catheter insertion for bladder emptying followed by immediate removal [3,4,5]. The safe execution of these techniques requires not only procedural knowledge, such as aseptic technique, anatomical understanding, and material handling, but also the ability to recognize early signs of complications and respond appropriately to clinical changes.

In home-based care, informal caregivers, often family members or close social contacts, play a pivotal role in managing urinary catheters, particularly in chronic conditions requiring long-term catheterization or clean intermittent catheterization. Importantly, formal and informal caregivers differ substantially in training, responsibilities, and scope of practice. While formal caregivers (e.g., nurses and other healthcare professionals) operate within regulated frameworks, with standardized training and clinical oversight, informal caregivers often rely on fragmented education, self-directed learning, and limited supervision. These differences have direct implications for the design, usability, and effectiveness of training interventions, particularly in the context of technically demanding procedures such as urinary catheterization [3,4,5,6].

However, evidence consistently shows that caregivers frequently experience substantial challenges, including insufficient technical knowledge, lack of confidence, and inadequate post-discharge support [6]. Epidemiological data suggest that the use of urinary catheters in community settings is both significant and under-recognized. In Brazil, approximately 2.63% of patients receiving home care use urinary catheters [2], while prevalence estimates in high-income countries vary by age and care setting, reaching over 1% among older adults and exceeding 7% in long-term care institutions [7,8]. In low- and middle-income settings, rates may be even higher, reflecting disparities in access to care and chronic disease burden [9]. Together, these data highlight the growing reliance on caregivers in managing complex procedures traditionally performed in clinical environments.

Despite their central role, caregiver training remains fragmented, inconsistent, and often insufficient. Transitional care processes are frequently characterized by information overload at discharge, lack of standardized educational pathways, and poor coordination across healthcare services [10,11]. Traditional educational approaches, such as verbal instructions and printed materials, are limited in their ability to ensure knowledge retention, skill acquisition, and correct procedural execution in real-world contexts, particularly among caregivers with low health literacy or limited prior experience with invasive procedures. This persistent gap reflects a broader research-to-practice divide, in which evidence-based caregiver training strategies are not effectively translated into routine care [12].

In recent years, mobile health (mHealth) technologies have emerged as promising tools to address these challenges by enabling continuous, accessible, and personalized learning. Interactive mobile applications, in particular, offer a combination of multimedia resources, step-by-step guidance, real-time feedback, and decision-support functionalities that align with principles of adult learning and behavior change. When grounded in theoretical frameworks and tailored to user characteristics, such as age, educational level, and digital literacy, these interventions have demonstrated potential to improve knowledge, practical skills, self-efficacy, and caregiving outcomes [13,14,15].

However, the effectiveness of these technologies is strongly influenced by users’ digital literacy and engagement capacity. Caregivers may face barriers such as limited familiarity with digital interfaces, difficulties in interpreting health information, and challenges in integrating app-based guidance into daily care routines [16,17,18]. Moreover, evidence suggests that while caregivers recognize the value of digital health tools, their actual use remains heterogeneous and dependent on usability, perceived usefulness, and contextual support [19].

Importantly, caregivers tend to prefer digital tools that provide real-time, actionable, and multidimensional support, including monitoring and guidance functionalities, rather than passive information delivery alone [19]. This highlights the need to move beyond static educational models toward more interactive and training-oriented solutions.

Within the specific context of urinary catheterization, emerging studies have explored the use of mobile applications as tools to support both patients and caregivers. Applications such as Punsook mobile application (Health Informatics Laboratory, Chiang Mai, Thailand), developed for individuals with spinal cord injury, and Participatient (Leiden University Medical Center—LUMC, Leiden, The Netherlands), designed to promote shared decision-making regarding catheter use, illustrate the potential of digital interventions to improve self-management and reduce inappropriate catheter use [20,21]. However, these initiatives remain heterogeneous in scope, functionality, and target populations, and are often not explicitly designed as structured training tools for caregivers.

Critically, the literature reveals a scarcity of training-focused digital interventions specifically designed to build caregiver competence. Most available mHealth tools emphasize monitoring or information provision rather than structured skill development, guided practice, and competency-based training, elements that are essential for effective caregiver-mediated care [12].

Despite the widespread use of urinary catheters, little is known about how interactive mHealth applications specifically support caregiver training in this context. To better conceptualize the role of mobile applications in this field, we propose a conceptual framework for mHealth-supported caregiver training in urinary catheterization, which integrates three interrelated domains:

(1) Educational components (e.g., structured content, multimedia modules, and microlearning strategies),
(2) Interactive functionalities (e.g., checklists, decision-support algorithms, reminders, and feedback mechanisms), and
(3) Implementation context (e.g., caregiver characteristics, health literacy, digital access, and integration with healthcare services).

These domains interact to influence proximal outcomes, such as knowledge acquisition, skill performance, and self-efficacy, which, in turn, may lead to distal outcomes including improved care quality, reduced complications, and decreased healthcare utilization. Importantly, this framework also recognizes cross-cutting factors such as usability, accessibility, data governance, and equity as critical determinants of successful implementation and scalability.

Despite the growing interest in digital health solutions, there remains a notable lack of comprehensive syntheses focusing specifically on interactive mobile applications designed to train caregivers in urinary catheterization. Existing literature is fragmented, often centered on patient education or disease-specific interventions, and rarely addresses the pedagogical and implementation dimensions of caregiver-focused technologies.

Therefore, this scoping review aims to map and synthesize the available evidence on the use of interactive mobile applications for caregiver training in urinary catheterization.

## 2. Materials and Methods

### 2.1. Study Design

This study is a scoping review conducted to map and synthesize the available evidence on the use of interactive mobile applications for caregiver training in urinary catheterization. The review was developed in accordance with the methodological framework proposed by the Joanna Briggs Institute (JBI) for scoping reviews [22] and is reported following the Preferred Reporting Items for Systematic Reviews and Meta-Analyses extension for Scoping Reviews (PRISMA-ScR) [23].

The review question was formulated using the PCC (Population–Concept–Context) framework, which is recommended for scoping reviews [22]. The population comprised formal and informal caregivers; the concept referred to interactive mobile applications incorporating features such as actionable checklists, decision-support flows, feedback systems, reminders, and multimedia resources; and the context involved training processes related to the assessment, management, and safe execution of urinary catheterization.

A protocol was developed a priori, defining the research question, eligibility criteria, search strategy, data extraction plan, and synthesis approach. Although protocol registration is considered best practice to enhance transparency, it is not mandatory for scoping reviews. In this study, the decision not to register the protocol was based on its exploratory nature and the absence of anticipated methodological deviations. To mitigate potential bias and ensure methodological rigor, all procedures were predefined and consistently applied throughout the review process.

### 2.2. Search Strategy and Information Sources

A comprehensive search strategy was designed to identify relevant studies across multiple databases. The following electronic databases were searched: PubMed/MEDLINE, Scopus, Web of Science, and LILACS. In addition, grey literature was explored through Google Scholar, with screening restricted the first 100 results sorted by relevance, as recommended for feasibility and yield optimization in scoping reviews [24].

The search strategy combined controlled vocabulary (e.g., MeSH terms) and free-text terms related to caregivers, mobile applications, and training/education, using Boolean operators (AND, OR) and field restrictions (title, abstract, and keywords). A representative search strategy included terms related to caregivers, mobile applications, education/training, and urinary catheterization. Search strategies were adapted to the syntax and indexing systems of each database, including equivalent terms in Portuguese and Spanish for LILACS. No restrictions were applied regarding publication date, and studies published in English, Portuguese, or Spanish were considered eligible.

The full search strategies for all databases are provided in Appendix A, ensuring transparency and reproducibility of the review process. The last search update was conducted in April 2026.

### 2.3. Eligibility Criteria

Studies were selected based on predefined inclusion and exclusion criteria aligned with the PCC framework. Eligible studies included original research (e.g., pilot studies, feasibility studies, quasi-experimental designs, randomized trials, and observational studies) that evaluated interactive mobile applications used by caregivers for training in urinary catheterization.

To be included, studies were required to: (i) involve formal or informal caregivers; (ii) evaluate mobile applications with interactive components (e.g., checklists, decision-support tools, feedback mechanisms, reminders, or multimedia features); and (iii) report at least one educational, behavioral, or implementation-related outcome.

Studies were excluded if they did not address caregiver training, educational interventions, or support strategies related to urinary catheter care. The exclusion criteria were defined to ensure alignment with the review objective, rather than based on professional scope (e.g., nursing-specific practice).

### 2.4. Study Selection

All retrieved records were exported in RIS or BibTeX format and managed using Rayyan. Available online: https://www.rayyan.ai/ (accessed on 13 August 2025). Duplicate records were removed through a two-step process: automated detection based on title, authors, year, and DOI, followed by manual verification of residual duplicates.

Study selection was conducted in two phases by two independent reviewers:

(1) title and abstract screening, followed by
(2) full-text assessment of potentially eligible studies.

Disagreements between reviewers were resolved through discussion and consensus, with arbitration by a third reviewer when necessary.

Inter-rater agreement was assessed during the screening process using Cohen’s kappa coefficient, providing a measure of consistency between reviewers [20]. Cohen’s kappa coefficient indicated substantial agreement (κ = 0.82). The agreement was considered acceptable based on established benchmarks for reliability.

**Figure 1:**
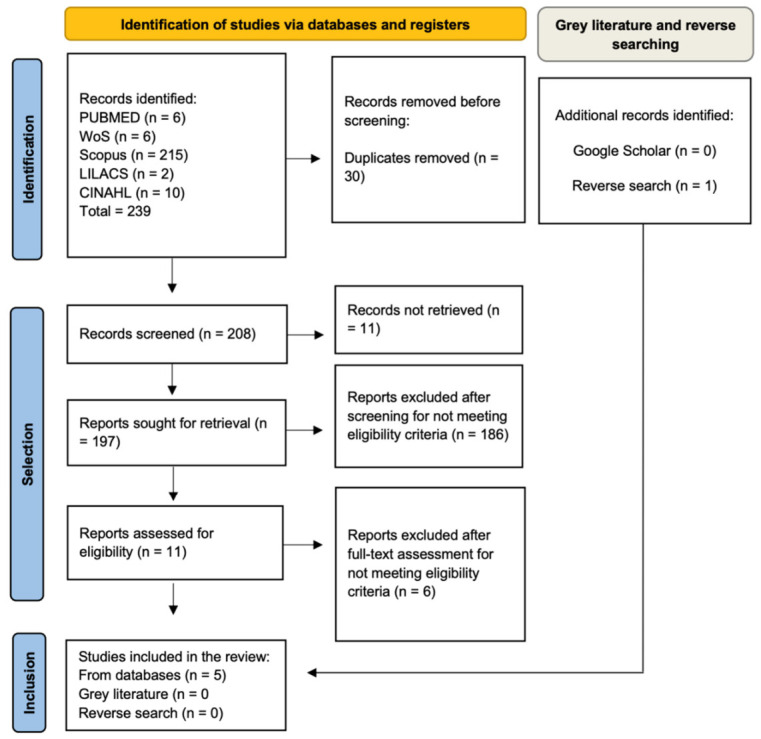
PRISMA flow diagram of study identification, screening, and inclusion.

The study selection process is presented in a PRISMA-ScR flow diagram, including the number of records identified, screened, assessed for eligibility, and included, as well as reasons for exclusion at the full-text stage (Figure 1).

A total of 239 records were identified, of which 208 remained after duplicate removal. One additional record was identified through reverse searching but did not meet eligibility criteria. Following screening, 11 full-text articles were assessed for eligibility, resulting in five studies included in the final sample.

### 2.5. Data Extraction

Data were extracted using a standardized and pilot-tested form developed by the research team. The extraction form captured the following information:

☐ Study identification (author, year, country);
☐ Study design and setting;
☐ Characteristics of the caregiver population (formal/informal; care context);
☐ Description of the intervention (platform, content, interactive features, integration with telemonitoring or health systems, duration, and mode of use);
☐ Educational outcomes (e.g., knowledge, skills, procedural performance, recognition of warning signs, retention);
☐ Implementation outcomes (e.g., usability, acceptability, engagement, adherence)
☐ Clinical and process outcomes (when available);
☐ Reported limitations and implementation barriers/facilitators.

Data extraction was performed independently by two reviewers to ensure accuracy and consistency. Discrepancies were resolved through consensus.

Missing or unreported data were explicitly labeled as “not reported,” and no data imputation was performed.

### 2.6. Data Synthesis

Data were synthesized using a combination of descriptive and narrative approaches, as recommended for scoping reviews [22]. A tabular synthesis was developed to summarize key characteristics of the included studies, the features of the mobile applications, and the reported outcomes.

The synthesis was structured into three main domains:

(1) study characteristics;
(2) intervention components and functionalities;
(3) outcomes, implementation aspects, and limitations.

A narrative synthesis was conducted to integrate findings across studies, identify patterns and divergences, and highlight key evidence gaps. Particular attention was given to the alignment between intervention components and reported outcomes, as well as to methodological limitations and implications for future research and practice. No critical appraisal was performed, as recommended for scoping reviews focusing on mapping evidence rather than assessing methodological quality [22].

## 3. Results

A total of five studies met the inclusion criteria of this scoping review, reflecting a limited and emerging evidence base on the use of interactive mobile applications for caregiver training in urinary catheterization. The included studies were published between 2020 and 2025 and conducted across three countries, Japan, Turkey, and Australia, highlighting a geographically restricted but internationally distributed body of research.

As summarized in Table 1, the methodological profile of the included studies was heterogeneous, encompassing development studies, qualitative research, mixed-methods protocols, quasi-experimental designs, and only one randomized controlled trial. Most studies were conducted in single-center settings and involved small sample sizes or did not report sample size, indicating early-stage investigations with limited generalizability.

The populations addressed varied considerably, including informal caregivers (predominantly parents of children undergoing clean intermittent catheterization), formal caregivers (such as visiting nurses), and mixed groups involving patients, caregivers, and healthcare professionals. The care settings ranged from home-based care to hospital and community contexts, reflecting the cross-sectoral nature of urinary catheter management.

Overall, the evidence is largely concentrated in exploratory, development, and implementation phases, with a predominance of feasibility-oriented studies and a scarcity of robust experimental designs. This distribution underscores the nascent stage of research in this field and points to important gaps in high-quality evidence evaluating the effectiveness of mHealth interventions for caregiver training.

The characteristics of the mHealth interventions and their core training components are summarized in Table 2. Across the included studies, mobile applications consistently combined structured educational content with interactive and behavioral features, reflecting a multimodal approach to caregiver training.

Educational components typically included step-by-step guidance on catheterization techniques, hygiene practices, and recognition of warning signs, often organized into modular or microlearning formats. In parallel, interactive functionalities, such as decision-support tools, case-based learning, and guided navigation, were designed to support real-time application of knowledge and reduce procedural uncertainty.

Behavioral features, including reminders, feedback mechanisms, and spaced learning strategies, were incorporated in several interventions to reinforce adherence and promote sustained engagement. Notably, some applications were explicitly grounded in theoretical frameworks, such as the Roy Adaptation Model or principles of microlearning and Appreciative Inquiry, suggesting an effort to align technological design with behavioral and educational theories.

Overall, the interventions demonstrate a convergence toward integrated digital training models that combine educational, interactive, and behavioral elements. However, variability remains in the depth of theoretical grounding, the sophistication of interactive features, and the extent to which behavioral components are systematically implemented.

The outcomes, effectiveness, and implementation aspects of the included studies are summarized in Table 3. Overall, the findings indicate that interactive mobile applications are feasible and potentially beneficial tools for caregiver training in urinary catheterization; however, the strength and scope of the evidence remain limited.

Educational outcomes were the most consistently reported across studies, with improvements observed in caregiver knowledge, technical skills, self-confidence, and coping or adaptation processes. These gains were primarily based on self-reported measures or short-term assessments, with limited use of objective or standardized evaluation tools.

Clinical outcomes were less frequently assessed, although two studies reported reductions in urinary tract infection incidence and, in one case, decreased antibiotic resistance among patients whose caregivers received app-based training. Despite these promising findings, clinical evidence remains scarce and is largely derived from small, single-center studies.

Implementation outcomes, including usability, feasibility, acceptability, and engagement, were commonly explored, particularly in development and qualitative studies. These findings highlight the perceived usefulness of mobile applications in supporting decision-making, standardizing care processes, and facilitating communication between caregivers and healthcare professionals.

Across studies, key limitations included small sample sizes, short follow-up periods, lack of robust comparators, and absence of standardized outcome measures. Notably, several studies did not assess clinical outcomes, reinforcing the gap between technological development and rigorous evaluation of effectiveness.

The alignment of the included studies with the conceptual framework proposed in this review is presented in Table 4. Overall, the findings demonstrate that most interventions incorporate educational and interactive components, while behavioral features are less consistently integrated.

Educational components were present across all studies, typically structured as protocol-based or stepwise training content aimed at supporting caregiver knowledge and procedural accuracy. Interactive components, such as decision-support tools, guided navigation, and case-based learning, were also widely implemented, suggesting an emphasis on supporting real-time application of knowledge during care delivery.

In contrast, behavioral components, including reminders, feedback, and reinforcement strategies, were less uniformly adopted, being more prominent in studies grounded in theoretical models or learning frameworks. This variability indicates that while technological and educational aspects are well represented, behavior change strategies remain underdeveloped in several interventions.

Regarding outcomes, most studies reported improvements in proximal outcomes, particularly caregiver knowledge, skills, and self-efficacy. However, distal outcomes, such as reductions in complications or healthcare utilization, were infrequently assessed and remain limited to a small number of studies.

Overall, this synthesis highlights a partial alignment between existing interventions and the proposed conceptual framework, with stronger emphasis on educational and interactive domains and notable gaps in behavioral integration and robust clinical evaluation.

**Figure 2:**
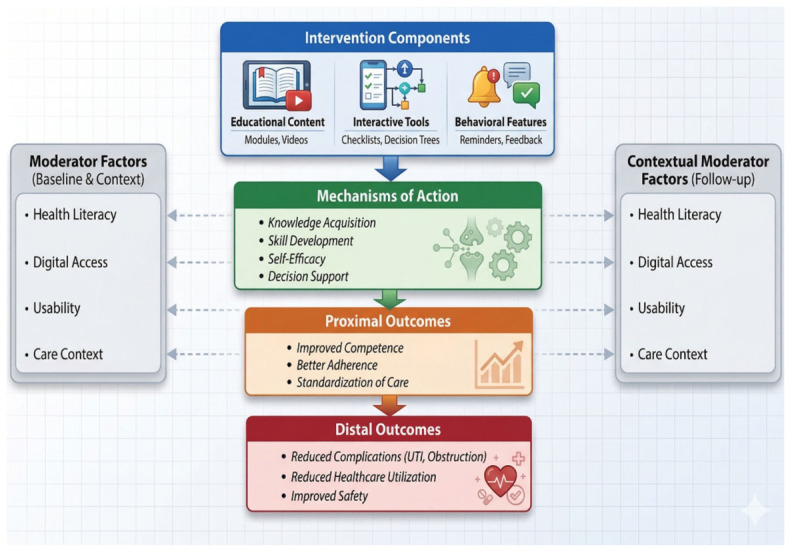
Conceptual framework of mHealth-supported caregiver training in urinary catheterization.

The integration of findings across the included studies enabled the development of a conceptual framework describing how mHealth interventions support caregiver training in urinary catheterization (Figure 2). This framework synthesizes the relationships between core intervention components, underlying mechanisms of action, and reported outcomes.

Across studies, interventions consistently combined educational content, interactive tools, and, to a lesser extent, behavioral features. These components appear to operate through key mechanisms, including knowledge acquisition, skill development, self-efficacy enhancement, and decision support, which collectively contribute to improvements in caregiver competence and adherence to recommended practices.

These proximal effects may, in turn, influence distal outcomes, such as reduction in catheter-related complications and improved patient safety, although such outcomes remain underexplored in the current evidence base. Additionally, contextual factors, such as health literacy, digital access, usability, and care setting, emerge as important moderators influencing the effectiveness and implementation of these interventions.

## 4. Discussion

This scoping review provides a synthesis of current evidence on interactive mHealth applications for caregiver training in urinary catheterization, highlighting a field that remains incipient but conceptually structured. Although only a small number of studies were identified, their convergence around similar intervention components suggests an emerging, yet coherent, model of digital support for caregiver education. This pattern aligns with broader trends in mHealth research, where initial developments tend to prioritize feasibility and usability before progressing toward more robust evaluations of clinical effectiveness [14,30,31].

Across the included studies, a consistent configuration of intervention components was observed, comprising educational content, interactive functionalities, and, to a lesser extent, behavioral features. Rather than acting independently, these elements appear to function synergistically. Educational components provide foundational knowledge and procedural guidance, while interactive features, such as decision-support tools and guided navigation, facilitate the real-time application of that knowledge. This integration is particularly relevant in home-based care contexts, where caregivers must perform complex tasks with limited professional supervision.

However, an important imbalance emerges within this configuration. While educational and interactive elements are well represented, behavioral components remain underdeveloped. Features such as reminders, feedback mechanisms, and reinforcement strategies were inconsistently implemented, despite strong evidence from other areas of digital health demonstrating their importance in sustaining behavior change and improving long-term outcomes [13,32,33]. This gap may help explain why most studies focused on short-term improvements in knowledge and skills, with limited evidence of sustained adherence or clinical impact.

These findings suggest that current mHealth interventions are primarily designed to support immediate task execution rather than long-term behavior change. In the context of urinary catheterization, a procedure that requires consistency, vigilance, and early recognition of complications, this limitation is particularly relevant. Effective caregiver support extends beyond knowledge acquisition to include ongoing reinforcement and decision-making support over time.

From a clinical perspective, the mechanisms identified across studies, knowledge acquisition, skill development, self-efficacy, and decision support, are closely aligned with core principles of nursing care. In this sense, mobile applications can be understood not as substitutes, but as extensions of nursing practice, functioning as cognitive and educational aids that enhance caregiver performance in real-world settings. This interpretation is consistent with evidence demonstrating that structured education and adherence to protocols are essential for preventing catheter-associated complications [34,35,36,37].

Despite these promising mechanisms, the evaluation of outcomes remains limited. Most studies assessed proximal outcomes, such as knowledge, skills, and self-efficacy, often using self-reported measures or short-term assessments. In contrast, distal outcomes, such as reduction in urinary tract infections or healthcare utilization, were rarely evaluated and, when reported, were based on small and context-specific samples. This imbalance reflects a broader challenge in mHealth research, where technological innovation frequently outpaces the generation of high-quality evidence on effectiveness and cost-effectiveness [14,30].

When compared to more mature domains of digital health, such as chronic disease management or remote monitoring, the interventions identified in this review appear less advanced in terms of personalization, system integration, and adaptive functionality. Many contemporary applications incorporate real-time data analytics, integration with electronic health records, and tailored feedback based on user behavior. In contrast, catheter-related applications remain largely centered on static or semi-interactive content. This gap highlights an important opportunity for future development, particularly in integrating these tools into telehealth systems and nursing workflows to enhance continuity of care.

Another critical dimension concerns health literacy and digital inclusion. Caregivers responsible for urinary catheter management are often required to interpret complex information and perform technically demanding tasks, frequently without adequate preparation or ongoing support [6,11]. Both health literacy and digital literacy play a central role in determining the effectiveness of mHealth interventions. The limited consideration of these factors in the included studies raises concerns about the potential for digital tools to exacerbate existing inequalities, particularly among older caregivers or those with limited access to technology [30,31].

At the system level, the implementation of mHealth interventions depends not only on individual engagement but also on organizational and structural conditions. Integration into care pathways requires alignment with clinical protocols, interoperability with health information systems, and clear delineation of professional responsibilities. Furthermore, issues related to data governance, privacy, and ethical use of health information remain insufficiently addressed in the current literature, representing an important gap for future research.

From a nursing perspective, these findings reinforce the central role of nurses in mediating the use of digital technologies in caregiver training. Nurses are uniquely positioned to assess caregiver needs, tailor educational strategies, and facilitate the safe and effective adoption of mHealth tools. Rather than replacing professional care, these technologies should be understood as complementary resources that extend the reach of nursing interventions beyond institutional settings and support continuity of care.

### 4.1. Implications for Nursing Practice

The findings of this review have direct and meaningful implications for nursing practice. First, they support the incorporation of interactive mobile applications as complementary tools in caregiver education, particularly in the context of hospital discharge and transitional care. These technologies can enhance the continuity of education, reinforce critical steps in catheter management, and provide real-time support for decision-making, thereby reducing uncertainty and improving the safety of care.

Second, nurses play a critical role in mediating the use of these technologies, ensuring that applications are appropriate for the caregiver’s level of health and digital literacy. This includes adapting communication strategies, selecting user-friendly tools, and providing ongoing support to promote effective engagement.

Third, the integration of mHealth tools into nursing practice offers opportunities to standardize care, reduce variability in technique, and improve adherence to evidence-based guidelines for catheter management and infection prevention. This is particularly relevant in light of the persistent challenges associated with catheter-associated urinary tract infections and the need to strengthen preventive strategies [34,35].

Finally, nurses should be actively involved in the development, evaluation, and implementation of digital health interventions, contributing their clinical expertise to ensure that these tools are safe, effective, and aligned with the realities of care. The development of competencies in digital health should therefore be considered a priority in nursing education and professional development, enabling nurses to lead and innovate in the integration of technology into patient care. This positions nurses not only as end-users but as key stakeholders in the design, validation, and governance of digital health interventions.

### 4.2. Limitations

This review should be interpreted in light of some limitations. First, the heterogeneity in how “interactivity” was conceptualized and operationalized across studies may have influenced study selection and synthesis. While this review adopted a structured definition aligned with decision-support, feedback, and actionable components, variations in terminology and reporting may have led to the exclusion of interventions with relevant but less explicitly described interactive features. Additionally, no formal methodological quality appraisal was conducted, as recommended for scoping reviews focused on evidence mapping. However, given the small number and heterogeneity of included studies, the findings should be interpreted with caution, as methodological rigor varied across studies.

Second, the rapid evolution of digital health technologies poses an inherent temporal limitation. Given the pace at which mobile applications are developed, updated, or discontinued, some interventions identified in the literature may no longer be available, while more recent or unpublished tools may not yet be captured in indexed databases. This dynamic landscape may affect the contemporaneity and applicability of the findings.

Third, the predominance of studies embedded in specific health systems and cultural contexts limits the transferability of findings to other settings, particularly low- and middle-income countries with different digital infrastructures, caregiver profiles, and models of home-based care. Importantly, contextual factors such as digital access, health literacy, and organization of nursing care are not consistently reported, which restricts deeper analysis of implementation feasibility across diverse environments.

Finally, although this review proposed a conceptual framework to synthesize findings, this model was derived inductively from a limited number of heterogeneous studies and has not yet been empirically validated. Therefore, it should be interpreted as a heuristic tool to guide future research and practice rather than a definitive explanatory model.

## 5. Conclusions

This scoping review mapped and synthesized the available evidence on the use of interactive mobile applications for caregiver training in urinary catheterization, suggesting that these interventions may represent a promising approach, although current evidence remains preliminary and limited, to support safer and more structured care in home and transitional settings.

The findings indicate that mHealth applications, particularly when incorporating educational, interactive, and behavioral components, have the potential to enhance caregiver competence, support self-efficacy, and assist real-time decision-making. However, these conclusions should be interpreted with caution, given the predominance of early-stage studies and the limited methodological robustness of the available evidence.

Importantly, the current literature reveals a substantial gap between the development of digital solutions and the generation of high-quality evidence regarding their clinical and economic impact. In particular, the scarcity of studies assessing distal outcomes, such as reductions in catheter-associated complications, healthcare utilization, and system-level effects, remains a critical limitation.

By integrating the available evidence into a conceptual framework, this review contributes to a more structured understanding of how mHealth interventions may operate in caregiver training. The findings highlight the potential importance of behavioral components and contextual factors, including health literacy, digital access, and usability, as key determinants of effectiveness.

Within nursing practice, interactive mobile applications may be considered as complementary tools that can extend the reach of educational support beyond institutional settings. However, their effective implementation depends on careful integration into care processes and alignment with caregiver needs and health system contexts.

Future research should prioritize more rigorous, theory-informed, and implementation-oriented study designs, with greater emphasis on clinical outcomes and long-term effectiveness. Strengthening this evidence base will be essential to determine the real impact of these technologies and to support their safe and equitable integration into nursing care.

## Figures and Tables

**Table 1:** Characteristics and methodological profile of included studies.

Study	Author (Year)	Country	Design	*n*	Caregiver Type	Population	Setting	Study Phase
1	Maeda et al. (2021) [25]	Japan	Development study	NR	Mixed	Patients + caregivers + professionals	Home care	Development
2	Sari & Kalyoncu (2025) [26]	Turkey	Prospective comparative	40	Informal	Children with CIC	Pediatric nephrology clinic	Quasi-experimental
3	Sari & Demirbay (2025) [27]	Turkey	RCT	40	Informal	Children under CIC + caregivers	University hospital	Experimental
4	Alex et al. (2022) [28]	Australia	Mixed-methods protocol	NR	Mixed	Patients + caregivers + nurses	Community & hospital	Implementation
5	Fukuda et al. (2020) [29]	Japan	Qualitative	26	Formal	Visiting nurses	Home care services	Exploratory

CIC = clean intermittent catheterization; RCT = randomized controlled trial; NR = not reported.

**Table 2:** Characteristics of mHealth interventions and training components.

Study	App/Platform	Educational Components	Interactive Features	Behavioral Features	Training Focus	Theoretical Basis
(1) [25]	Multi-version catheter app	Protocol-based content	Decision flows, data sharing	Communication alerts	Occlusion prevention	Protocol-driven
(2) [26]	RAMACIC	Hygiene, technique, symptoms	Structured modules	Reminders, follow-up	CIC training	Roy Adaptation Model
(3) [27]	RAMTAKE	Stepwise training	Interactive modules	Feedback	Skills + coping	Roy Adaptation Model
(4) [28]	GoShare + QStream	Microlearning content	Case-based learning	Spaced repetition	Self-management	Appreciative Inquiry
(5) [29]	Protocol app	Protocol guidance	Stepwise navigation	Limited	Blockage prevention	Protocol-based

**Table 3:** Outcomes, effectiveness, and implementation aspects of included studies.

Study	Educational Outcomes	Clinical Outcomes	Implementation Outcomes	Key Findings	Limitations
(1) [25]	Not assessed	Not assessed	Feasibility	Improved coordination	No clinical validation
(2) [26]	Improved competence	Reduced UTI	Adherence	Reduced UTI long-term	Small sample
(3) [27]	Increased knowledge/skills	Reduced UTI	High acceptability	Effective intervention	Short follow-up
(4) [28]	Expected improvement	Not reported	Engagement	Potential benefits	Protocol only
(5) [29]	Not assessed	Not assessed	Usability	Design insights	No outcomes

**Table 4:** Alignment of included studies with the conceptual framework.

Study	Educational Components	Interactive Components	Behavioral Components	Proximal Outcomes	Distal Outcomes
Maeda et al. (2021) [25]	Protocol-based content for catheter management	Decision-support flows; data-sharing interface	Communication prompts between users	Not assessed	Not assessed
Sari & Kalyoncu (2025) [26]	Structured CIC training (technique, hygiene, symptom recognition)	Guided modules and structured navigation	Reminders and follow-up monitoring	Improved adherence and caregiver competence	Reduced UTI incidence and antibiotic resistance
Sari & Demirbay (2025) [27]	Stepwise skill-based training and coping strategies	Interactive learning modules	Feedback and reinforcement mechanisms	Increased knowledge, skills, and adaptation	Reduced UTI incidence
Alex et al. (2022) [28]	Digital educational content based on microlearning	Case-based interactive platforms	Spaced repetition and reminders	Increased knowledge and self-efficacy (expected)	Potential reduction in complications (not yet evaluated)
Fukuda et al. (2020) [29]	Protocol-oriented guidance for catheter management	Stepwise navigation support	Limited (prototype stage)	Perceived usefulness and usability	Not assessed

## Data Availability

Data sharing is not applicable. No new data were created or analyzed in this study.

## Public Involvement Statement

No public or patient involvement was associated with this research.

## Guidelines and Standards Statement

This study was conducted and reported in accordance with the Joanna Briggs Institute (JBI) methodology for scoping reviews and the Preferred Reporting Items for Systematic Reviews and Meta-Analyses extension for Scoping Reviews (PRISMA-ScR).

## Use of Artificial Intelligence

Artificial intelligence tools were used exclusively to support language editing and improve clarity of the English text. These tools did not contribute to data extraction, analysis, interpretation of results, or content generation. The authors take full responsibility for the integrity and accuracy of the manuscript. Protocol registration link: https://osf.io/zqknb/overview, (accessed on 12 May 2026).

## Appendix A

**Database**	**Search Date**	**Search Strategy**	**Records Retrieved**
PubMed/MEDLINE	17 April 2026	(“Caregivers”[Mesh] OR caregiver*[tiab] OR “family caregiver*”[tiab] OR “informal caregiver*”[tiab]) AND (“Mobile Applications”[Mesh] OR “mobile app*”[tiab] OR smartphone*[tiab] OR “mHealth”[tiab] OR “digital health”[tiab]) AND (“Education”[Mesh] OR training[tiab] OR education[tiab] OR “health education”[tiab] OR “patient education”[tiab]) AND (“Urinary Catheterization”[Mesh] OR “catheterization”[tiab] OR “catheterisation”[tiab] OR catheter*[tiab] OR “urinary catheter*”[tiab] OR “intermittent catheterization”[tiab])	6
Scopus	21 March 2026	TITLE-ABS-KEY((caregiver* OR “family caregiver*” OR “informal caregiver*”) AND (“mobile application*” OR “mobile app*” OR smartphone* OR mHealth OR “digital health”) AND (education OR training OR “health education” OR “patient education”) AND (“urinary catheter*” OR catheterization OR catheterisation OR “intermittent catheterization”))	215
Web of Science	21 March 2026	(caregiver* OR “family caregiver*” OR “informal caregiver*”) AND (“mobile application*” OR “mobile app*” OR smartphone* OR mHealth OR “digital health”) AND (education OR training OR “health education”) AND (catheterization OR catheterisation OR catheter* OR “urinary catheter*”)	4
LILACS	21 March 2026	((mh:“Cuidadores” OR caregiver* OR cuidadores OR cuidador)) AND ((mh:“Aplicativos Móveis” OR “aplicativo móvel” OR “mobile app*” OR smartphone OR mHealth)) AND ((mh:“Educação” OR treinamento OR education OR training)) AND ((mh:“Cateterismo” OR catheter* OR cateter*))	2
CINAHL	21 March 2026	(caregiver* OR “family caregiver*” OR “informal caregiver*”) AND (“mobile application*” OR “mobile app*” OR smartphone* OR mHealth OR “digital health”) AND (education OR training OR “health education”) AND (catheter* OR catheterization OR catheterisation OR “urinary catheter*”)	10
Google Scholar	21 March 2026	(caregiver OR caregivers) AND (“mobile application” OR “mobile app” OR smartphone OR mHealth) AND (education OR training) AND (catheterization OR catheter)	First 100 results screened; -no additional studies included
Note: The asterisk (*) was used as a truncation operator in the databases to encompass morphological variations in search terms.			
